# Editorial: Down Syndrome, Neurodegeneration and Dementia

**DOI:** 10.3389/fnagi.2021.791044

**Published:** 2021-12-09

**Authors:** Elliott J. Mufson, Stephen D. Ginsberg, Tao Ma, Aurélie Ledreux, Sylvia E. Perez

**Affiliations:** ^1^Department of Translational Neurobiology, Barrow Neurological Institute, Phoenix, AZ, United States; ^2^Center for Dementia Research, Nathan Kline Institute, Orangeburg, NY, United States; ^3^Department of Psychiatry, New York University Grossman School of Medicine, New York, NY, United States; ^4^Department of Neuroscience and Physiology, New York University Grossman School of Medicine, New York, NY, United States; ^5^NYU Neuroscience Institute, New York University Grossman School of Medicine, New York, NY, United States; ^6^Department of Internal Medicine-Gerontology and Geriatric Medicine, Wake Forest University School of Medicine, Winston-Salem, NC, United States; ^7^Department of Physiology and Pharmacology, Wake Forest University School of Medicine, Winston-Salem, NC, United States; ^8^Department of Neurobiology and Anatomy, Wake Forest University School of Medicine, Winston-Salem, NC, United States; ^9^Knoebel Institute for Healthy Aging, University of Denver, Denver, CO, United States; ^10^Department of Neurosurgery, University of Colorado Anschutz Medical Campus, Aurora, CO, United States

**Keywords:** Down syndrome, dementia, pathology, animal models, neurobiology, neurodegeneration, trisomy

The Cornish physician John L. Down published a paper entitled “Observations on an ethnic classification of idiots” (Down, 1866) (Figure 1), describing a condition referred to as a “mongoloid idiot.” Interestingly, Down's grandson was born with this condition (Figure 1) (Salehi et al., 2016). Down assumed that parental tuberculosis caused this disorder (Van Robays, 2016). However, almost a century later, genetic analysis by Lejeune, Gautier and Turpin (Lejeune et al., 1959) revealed that this syndrome was due to an extra copy of chromosome 21 (HSA21) (Figure 1), which encodes the gene for amyloid-beta precursor protein (APP). In 1965, the World Health Organization confirmed the eponym for this disorder as Down syndrome (DS). The discovery of the gene that encodes the APP protein, which includes the beta-amyloid (Aβ) peptide, and that resides on chromosome 21 was first reported by Goldgaber (Goldgaber et al., 1987) followed by other published works (Kang et al., 1987; Robakis et al., 1987; Watkins et al., 1987; Korenberg et al., 1989). Trisomy 21 leads to an overproduction of the Aβ peptide associated with DS (Glenner and Wong, 1984), AD (Wisniewski et al., 1988), and familiar AD (FAD) (Teller et al., 1996; Russo et al., 1997; Mori et al., 2002). It is interesting to note that several genes on chromosome 21 have been associated with cognitive dysfunction in DS, however, the APP gene alone is necessary and sufficient to cause dementia (Doran et al., 2017). Recently, it was reported that DS affects approximately 200,000 people in the US and 5–8 million worldwide (de Graaf et al., 2017). Interestingly, there is an age-associated clinical and pathological coexistence between DS and AD, which is a major public health issue. Life expectancy of people with DS has increased dramatically over the past decades (from 25 years in the 1980s to 60+ years currently) and consequently age-related cognitive syndromes have also increased (Ruparelia et al., 2013; Godfrey and Lee, 2018). However, the neurobiology underlying the onset of dementia in individuals with DS remains a complex question. Individuals with DS develop selective neuronal degeneration, synaptic loss, neurofibrillary tangles, and Aβ plaques similar to AD (Mirra et al., 1991; Hyman and Trojanowski, 1997) by the fourth decade of life (Mann et al., 1989; Hartley et al., 2015) and is now recognized as a genetically-determined form of AD (Fortea et al., 2020).

**Figure 1:**
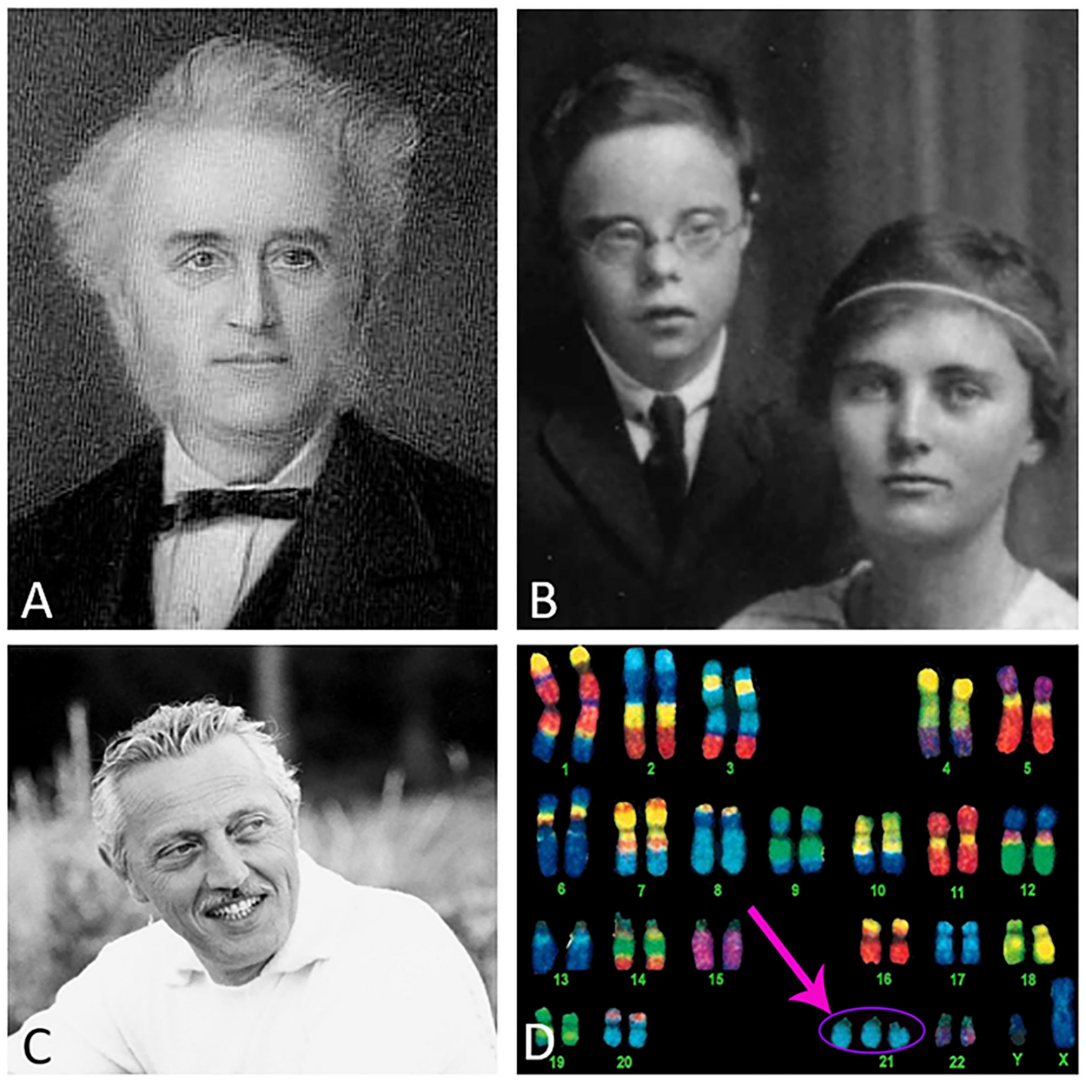
Dr. Down **(A)**, Down's grandson and daughter **(B)**, Dr. Lejeune **(C)**, and an image of a Down syndrome karyotype indicating the extra copy of chromosome 21 (pink arrow) **(D)**. Image credits: **(A)** Wikipedia, **(B)** photo courtesy of Global Down Syndrome Foundation, **(C)** reproduced with permission from the Jerome Lejeune Foundation, and **(D)** @prayersforbabyfinn webpage.

Approximately 70% of people with DS >50 years of age display dementia, which may be an underestimate. Despite DS being the largest group of individuals with early-onset AD, there is a lack of knowledge defining the mechanisms driving neuronal and functional dysfunction in both disorders, impeding drug discovery. Importantly, the prevalence of AD in DS makes it possible to enroll this population in clinical trials. Although not totally representative of either disorder, mouse models recapitulate key aspects of DS and AD, enabling the assessment of pathophysiological mechanisms (Reeves et al., 1995; Li et al., 2007; Haydar and Reeves, 2012). The current Research Topic “Down syndrome, Neurodegeneration and Dementia” highlights basic and translational research in DS. In total, seven manuscripts evaluated human DS and three reports studied murine models of DS and related AD pathobiology.

Chen et al. discuss products of triplicated genes on HSA21 that may modify the effect of APP in DS related to endosomal-lysosomal, neurotrophin, axonal transport, and immunological cellular systems that affect people with DS that go on to contract Covid-19.

Pivtoraiko et al. discuss the interaction between Pittsburgh Compound B (PiB), or related amyloid binding radiopharmaceuticals for positron emission tomography (PET) imaging, with different unmodified Aβ forms or post-translationally truncated and pyroglutamate-modified Aβ in adults with DS and AD. Despite the distinct molecular profile of Aβ forms and greater vascular amyloidosis in DS, cortical ^3^H-PiB binding does not distinguish between groups at an advanced level of amyloid plaque pathology suggesting differences in pathobiological mechanism(s) driving dementia.

Ahmed et al. suggest that the innate immune system activator granulocyte-macrophage colony-stimulating factor (GM-CSF) may have a therapeutic and/or compensatory action in animal models of DS, AD, and normal aging. They argue that in AD clinical trials activating the innate immune system may have paradoxical effects, and that inflammation may be therapeutic rather than deleterious.

Martinez et al. review the role of basal forebrain cholinergic (BFC) neuronal function and degeneration in AD and DS and identify under-studied aspects of BFC neuronal biology. Cuello and coworkers (Do Carmo et al.) review mechanisms underlying the compromise of the neurotrophin, nerve growth factor (NGF) in AD and DS. Similarities between dysfunction in the NGF neurotrophic system suggests that drugs related to the preservation of this neurotrophic pathway are treatment approaches for both DS and AD.

The Mufson group (Miguel et al.) examined the effect of trisomy on amyloid, Purkinje cells (PC), and interneurons within the cerebellum in DS. Their findings suggest that disturbances in calcium binding proteins play a critical role in cerebellar neuronal circuit dysfunction in adults with DS. The data suggests that drugs targeting specific calcium binding proteins are a novel target to prevent cerebellar cellular degeneration, which could impact cognition in DS.

Wang et al. explored sex-related genetic heterogeneity in AD by investigating single nucleotide polymorphism (SNP) heritability, genetic correlation, as well as SNP- and gene-based genome-wide analyses. The authors indicate an overall similar genetic architecture of AD in both sexes at the genome-wide averaged level and that clinically observed sex differences arise from sex-specific variants. This observation is important for the development of personalize medicine.

The article by the Ginsberg laboratory (Alldred et al.) investigated dysregulation of genes and encoded proteins of the oxidative phosphorylation pathway within the basocortical projection system in young Ts65Dn mice. The authors suggest that dysregulation within mitochondrial oxidative phosphorylation complexes is an early marker of basocortical degeneration in DS. These findings indicate a crucial role for alterations of oxidative gene expression as a potential avenue for future treatment approaches for DS with translation to AD.

The Velazquez group (Winslow et al.) discusses the use of the novel IntelliCage behavioral testing apparatus to overcome pervasive animal handling issues that occur during cognitive testing using the well-established 3xTg-AD animal model. The authors demonstrate deficits in cognition in the 3xTg-AD mouse and provide important factors to consider when testing models of AD and DS in the IntelliCage. These findings suggest that this novel technology is an important new tool for the investigation of cognitive deficits in animal models of dementia.

Strupp and coworkers (Powers et al.) present new evidence that dietary maternal choline supplementation during pregnancy and lactation has beneficial effects on cognition in young and old Ts65Dn mice throughout life, suggesting that this nutritional supplement would have population-wide benefits and provide an early intervention for DS fetuses.

A general comment about the Research Topic: Down syndrome, Neurodegeneration and Dementia. It is difficult to include all aspects of basic, translational, and clinical research related to DS in the context of a series of a dedicated papers. Rather, a tacit goal of the Research Topic in *Frontiers in Aging Neuroscience* is to increase overall interest in this underserved area of research and bring new investigators from other fields that will use *in vivo* and *in vitro* models of DS and AD. We also encourage studies using clinically and neuropathologically well-characterized tissue from human DS brain repositories to further provide therapeutic development that will assist this very special population of individuals as well as drug and treatment discovery for AD dementia and related disorders.

## Author Contributions

EM drafted the manuscript. SG, TM, AL, and SP edited the manuscript. All authors take responsibility for the integrity of the data and the accuracy of the data presented in the article. All authors contributed to the article and approved the submitted version.

## Funding

This work was supported by National Institute of Health grants P01 AG014449, R01 AG061566 (EM), P01 AG017617 (SG), R01 AG055581, R01 AG056622, R01 AG073823 (TM), Arizona Alzheimer's Disease Consortium at Barrow Neurological Institute (SP), Barrow Neurological Foundation (SP), and BrightFocus Foundation and Fein Foundation (EM). The funders had no role in the study design, data collection and analysis, decision to publish, or preparation of the manuscript.

## Conflict of Interest

The authors declare that the research was conducted in the absence of any commercial or financial relationships that could be construed as a potential conflict of interest.

## Publisher's Note

All claims expressed in this article are solely those of the authors and do not necessarily represent those of their affiliated organizations, or those of the publisher, the editors and the reviewers. Any product that may be evaluated in this article, or claim that may be made by its manufacturer, is not guaranteed or endorsed by the publisher.
